# Hospital burden of coronary artery disease: Trends of myocardial infarction and/or percutaneous coronary interventions in France 2009–2014

**DOI:** 10.1371/journal.pone.0215649

**Published:** 2019-05-02

**Authors:** Jessica Pinaire, Jérôme Azé, Sandra Bringay, Guillaume Cayla, Paul Landais

**Affiliations:** 1 UPRES EA 2415, Clinical Research University Institute, Montpellier University, Montpellier, France; 2 LIRMM, UMR 5506, Montpellier University, Montpellier, France; 3 AMIS, Paul Valéry University, Montpellier, France; 4 Cardiology Department, Nîmes University Hospital, Montpellier University, Nîmes, France; Azienda Ospedaliero Universitaria Careggi, ITALY

## Abstract

**Background:**

Currently, cardiovascular disease (CVD) is widely acknowledged to be the first leading cause of fatality in the world with 31% of all deaths worldwide and is predicted to remain as such in 2030. Furthermore, CVD is also a major cause of morbidity in adults worldwide. Among these diseases, the coronary artery disease (CAD) is the most common cause, accounting for over 40% of CVD deaths. Despite a decline in mortality rates, the consequences of more effective preventive and management programs, the burden of CAD remains significant. Indeed, the rise in the prevalence of modifiable risk factors due to changes in lifestyle and health behaviors has further increased the burden of this epidemic. Our objective was to evaluate the hospital burden of CAD via MI trends and Percutaneous Coronary Intervention (PCI) in the French Prospective Payment System (PPS).

**Methods:**

MI/PCI were identified in the national PPS database from 2009 to 2014 for patients aged 20 to 99, living in metropolitan France. We examined hospitalisation, readmission and mortality trends using standardised rates.

**Results:**

Over the six-year period, we identified 678,021 patients, representing 900,121 stays of which, 215,224 had a MI and a PCI. Admission trends increased by nearly 25%. Acute MI cases increased every year, with an alarming increase in women, and more specifically in young women. Men were 3 times more hospitalised than women, who were older. A North-South divide was noted. Twenty seven percent of patients experienced readmission within 1 month. Trajectories of care were significantly different by sex and age. Overall in-hospital death was 3.3%, decreasing by 15% during the period. The highest adjusted mortality rates were observed for inpatients aged <40 or >80.

**Conclusion:**

We outlined the public health burden of this condition and the importance of improving the trajectories of care as an aid for better care.

## Introduction

Around the world, cardiovascular disease (CVD) is recognized as the leading cause of death (accounting for approximately 31% of all deaths worldwide) and is predicted to remain as such in 2030 [1]. Moreover, this is reinforced with by the World Health Organization (WHO), which forecasts an 11% increase in the burden of CVD by 2030, bringing the worldwide number of myocardial infarctions (MI) and stroke to approximately 36.2 million [2]. In addition, CVD was also a major cause of morbidity in adults worldwide during the 20th century. Today, people with MI have a risk of recurrence and/or development of coronary heart disease-related conditions six times higher than those with no history of MI [3]. Among these diseases, the coronary artery disease (CAD) is the most common cause, accounting for more than 40% of CVD deaths (Source WHO). Despite improvements in care, CAD remain among the top causes of disease, disability and death worldwide leading to a high consumption of health resources [4].

In France, in 2012, the crude hospitalization rates for ischaemic heart disease, acute coronary syndromes and MI were 33.9, 18.8 and 9.4 per 10^4^, respectively [5]. In 2011, standardized mortality rates in ischaemic heart disease were evaluated at 4.8 per 10^4^ [5]. Local and regional or even national analyses from register data, like WHO-MONICA Project, highlighted a decline of these rates in the ten last years [6–9]. However, they also showed a societal change placing women on equal terms with men for risk of MI occurrence and mortality. In parallel, a study related to the economical burden of MI after a first episode underlined that the cost of health care following a MI, has been multiplied by three over the last decade [10].

As MI treatment is performed in a health facility, it is possible to trace the patient pathways through the national hospital Prospective Payment System (PPS), using comprehensive hospital databases, regularly collected for billing purposes. Although these data are sometimes criticised due to inaccuracy and lack of completeness [11, 12], they are increasingly used for epidemiological studies [13]. Moreover, in France, the gradual increase in fee-for-service has enhanced the coding quality [14]. Our objective was to evaluate the hospital burden of CAD through the MI, Percutaneous Coronary Interventions (PCI), and other ischaemic heart diseases (OIHD) annual trends as well as the related costs using the French PPS data over a six-year period.

## Materials and methods

### Materials

The Agence Technique de l’Information Sur l’Hospitalisation (ATIH) waived the need for consent according to the Enforcement (decree No. 94-666). Since 1996, all French hospitals caring for medical and surgical patients have submitted anonymized patient data to the French Hospital Discharge Database (FHDD). Each discharge summary submitted to the FHDD is linked to a national grouping algorithm leading to a French diagnosis related group [15]; thereby allowing patient comorbidities to be recorded and linked [16]. The study was conducted according to the approval given by the ATIH. Authorization was also obtained from the Commission Nationale de l’Informatique et des Libertés (agreement No. 1375062). The data provided were de-identified.

#### MI/PCI case identification algorithm in the FHDD

Usually, authors identify acute ischaemic heart events by the principal discharge diagnosis code of acute coronary syndrome using the International Classification of Diseases Tenth Revision (ICD-10) codes [17–20]. The sensitivity has been evaluated to be around 76% [21]. We added criterion based on Percutaneous Coronary Intervention (PCI) (see S1 Table). The list of ICD-10 codes as well as those of the French Common Classification of Medical Procedures (CCMP) [22] used to detect a MI are referenced in S1 Table. The international definition of MI is based on the elevation of troponin, but there is no straightforward translation of the definition into ICD-10 codes. ST-elevation MI (STEMI) is the default for unspecified term “acute MI” (AMI) and Non-ST-elevation MI (NSTEMI) requires more documentation such as the procedures peformed. Thus, ICD-10 codes are insufficient to distinguish the type of MI (STEMI or NSTEMI) [23]. Consequently, we will differentiate AMI coded by I21, which corresponds to the acute type 1 MI (Q-wave, STEMI, NSTEMI) versus the other ischaemic heart diseases (OIHD), I22 to I24 ICD-10 codes, and the PCIs as well, presented in S1 Table.

#### Inclusion criteria

We used the identification algorithm described above to select hospital stays between March 1, 2009, and December 31, 2014, in metropolitan France for all patients aged 20 to 99, and living in metropolitan France. Datamanagement was performed to validate that hospital stays were consistent particularly in terms of gender, age and death.

#### Non-inclusion criteria

We did not include patients aged < 20, patients living in French overseas territories and ACS occurring during a hospital stay.

### Statistical analysis

We analysed the data according to first admission during the observation period, as well as hospitalisations, readmissions and in-hospital deaths by type of condition. We compared the AMI, OIHD and PCI only groups, as well as the AMI and non-AMI groups (combining OIHD and PCI only). In some cases, we also presented the results in the general case (MI/PCI) without distinguishing between groups.

#### Primary and associated diagnoses

We assessed incident cases according to the first diagnosis (AMI, OIHD, or PCI only) through the study period from 2010 to 2014. We calculated the global variation rate over the study period by taking into account every annual variation rate. We considered data according to sex. We estimated the average population as of 1st January of each year from data provided by the National Institute for Statistics and Economic Studies (https://www.insee.fr/fr/information/2008354). We then assessed standardised rates on age and sex using the direct standardisation method. The structure of the French metropolitan population of the current year was the reference. We analysed the health of the studied population by investigating their hospitalisation history and related diagnoses based on criteria used in Elixhauser score [24].

#### Burden and trends of MI/PCI hospitalisations

Specific annual hospitalisation rates were determined by reporting the number of hospitalisations in a given year to the average population by age group and sex for the same year. We assessed standardised rates with the same method as described above. Rates were examined by departments. We compared length of stays according to demographic and diagnosis criteria. The evolution of economic burden was estimated by calculating the hospitalisation costs based on the tariffs provided by the Ministry of Health. We used the Chi-squared test, Student test or ANOVA, as appropriate, to look for between-group statistical differences.

Since our objective was to evaluate the health care burden associated with MI/PCI, in this report the statistical unit was the stay, and we took into account all stays without distinguishing first episode from recurrences.

#### Burden and trends of MI/PCI readmissions

We considered readmission for an MI diagnosis or a PCI (see S1 Table). Readmission rates following an initial event (MI or PCI) were analysed using a Kaplan-Meier model according to demographic characteristics. Subgroups were compared with a log-rank test. We estimated duration until readmission. We performed the analysis of the principal diagnosis sequences in order to describe the care pathways and compare difference between groups with the Levene test [25].

#### Burden of in-hospital mortality

We reported the numbers of in-hospital deaths and estimated the mortality rates (gross and standardised) by age and sex, in relationship with the target population. Since the cause of death is not known from these data, this analysis was done in the general MI/PCI case without distinguishing the AMI and non-AMI groups.

All analyses were performed using R software [26] and Microsoft SQL Server Management Studio, version 10.0.1600.22.

## Results

We identified 900,121 stays representing 678,021 patients with MI during the study period. Nearly 45% of patients presented with AMI and 7% were OIHD. The remaining 48% of patients, they had a PCI (S1 Fig). Angioplasty was performed in 72% of AMI and 60% OIHD patients, respectively.

### Primary and associated diagnoses

#### Incidence case

Table 1 present the evolution of incident cases from 2010 to 2014. The distribution of incident cases by type of principal diagnosis showed an increase in AMI between 2010 and 2014 for both men and women. In contrast, the number of incident cases decreased for OIHD. About seventy percent of incident AMI cases and eighty percent of incident PCI cases were men.

**Table 1:** Annual incident numbers and standardised rates of hospitalised patients from 2010 to 2014 in metropolitan France for the first admission with the overall rate evolution. AMI: Acute Myocardial Infarction (ICD-10 code: I21)—OIHD: Other Ischaemic Heart Diseases (ICD-10 codes: I22 to I24)—PCI only i.e without AMI or OIHD.

	2010	2011	2012	2013	2014	Global rate (%)
Number of patients*						
**AMI**						
Men	34,557	34,672	36,451	37,053	37,881	9.6
Women	16,358	16,238	16,640	16,408	17,086	4.5
**OIHD**						
Men	5,813	6,393	5,734	5,618	5,074	-12.7
Women	2,278	2,414	2,191	2,040	1,923	-15.5
**PCI only**						
Men	51,135	49,466	51,095	51,410	54,636	6.8
Women	15,569	15,439	15,969	15,907	17,162	10.2
**Global**	115,570	113,043	115,223	114,270	118,418	2.4
Incident hospitalisation standardised rates* (per 10^4^)						
**AMI**						
Men	15.3	15.3	16.0	16.2	16.4	7.1
Women	6.6	6.5	6.6	6.5	6.7	1.5
**OIHD**						
Men	2.6	2.8	2.5	2.5	2.2	-15.4
Women	0.9	0.1	0.9	0.8	0.8	-11.1
**PCI only**						
Men	22.7	21.8	22.4	22.4	23.7	4.4
Women	6.3	6.2	6.4	6.3	6.8	7.8
**Global**	24.4	23.7	24.1	23.7	24.5	0.4

* First admission in French metropolitan population.

#### Related diagnoses

Hypertension, congestive heart failure and cardiac arrhythmia were among the most common comorbid conditions in both men and women. However, considering the age group, the most common comorbid condition was hypertension (see Table 2). We found high proportions in diabetes and obesity. There were significant proportions of diabetes and obesity. In addition, alcohol abuse affected about 7% of men up to 65 years old. Among the most common comorbidities included depression in women, weight loss and renal failure in the elderly, and solid tumor without metastasis in men >65 years old.

**Table 2:** Medical conditions of the population by sex and age group over 2009-2014.

	<45 years		45-65 years		65-85 years		>85 years	
	Men	Women	Men	Women	Men	Women	Men	Women
Medical conditions	n(%)	n(%)	n(%)	n(%)	n(%)	n(%)	n(%)	n(%)
Congestive heart failure	6,041 (24.2)	1,328 (26.0)	65,006 (31.2)	12,587 (30.1)	102,816 (44.8)	45,585 (44.9)	18,568 (63.6)	22,953 (78.7)
Cardiac arrhythmia	3,534 (14.2)	770 (15.1)	36,270 (17.4)	6,572 (15.7)	81,434 (35.5)	33,798 (33.3)	16,320 (55.9)	18,044 (61.8)
Valvular disease	276 (1.1)	102 (2.0)	3,617 (1.7)	866 (2.1)	10,618 (4.6)	4,990 (4.9)	2,138 (7.3)	2,151 (7.4)
Pulmonary circulation disorders	217 (0.9)	96 (1.9)	3,383 (1.6)	966 (2.3)	9,698 (4.2)	5,892 (5.8)	2,277 (7.8)	3,053 (10.5)
Peripheral vascular disorder	2,131 (8.5)	508 (10.0)	34,069 (16.4)	6,055 (14.5)	56,336 (24.6)	18,606 (18.3)	7,154 (24.5)	6,218 (21.3)
Hypertension (uncomplicated)	6,134 (24.6)	1,498 (29.4)	100,056 (48.1)	22,197 (53.1)	154,307 (67.3)	72,952 (71.8)	20,547 (70.4)	27,306 (93.6)
Hypertension (complicated)	430 (1.7)	132 (2.6)	6,878 (3.3)	1,818 (4.4)	15,475 (6.7)	8,512 (8.4)	2,897 (9.9)	4,048 (13.9)
Paralysis	236 (1.0)	71 (1.4)	2,905 (1.4)	623 (1.5)	6,237 (2.7)	3,010 (3.0)	1,099 (3.8)	1,562 (5.4)
Other neurologic disorders	623 (2.5)	192 (3.8)	5,462 (2.6)	1,260 (3.0)	11,127 (4.9)	5,342 (5.3)	2,163 (7.4)	2,703 (9.3)
Chronic pulmonary diseases	852 (3.4)	342 (6.7)	16,915 (8.1)	4,048 (9.7)	31,998 (14.0)	10,532 (10.4)	4,580 (15.7)	3,652 (12.5)
Diabetes	1,786 (7.2)	562 (11.0)	36,196 (17.4)	8,456 (20.2)	58,751 (25.6)	25,878 (25.5)	5,631 (19.3)	6,789 (23.3)
Hypothyroidism	200 (0.8)	225 (4.4)	3,431 (1.7)	3,460 (8.3)	7,661 (3.3)	12,335 (12.1)	1,471 (5.0)	4,309 (14.8)
Renal failure	468 (1.9)	143 (2.8)	8,079 (3.9)	1,929 (4.6)	30,467 (13.3)	12,762 (12.6)	8,209 (28.1)	8,015 (27.5)
Liver disease	485 (1.9)	101 (2.0)	5,205 (2.5)	935 (2.2)	5,088 (2.2)	1,895 (1.9)	429 (1.5)	438 (1.5)
Peptic ulcer disease (excluding bleeding)	118 (0.5)	18 (0.4)	1,666 (0.8)	322 (0.8)	2,485 (1.1)	1,058 (1.0)	322 (1.1)	303 (1.0)
AIDS/HIV infection	189 (0.8)	42 (0.8)	1070 (0.5)	114 (0.3)	246 (0.1)	27 (0.03)	7 (0.02)	1 (0.0)
Lymphoma	89 (0.4)	32 (0.6)	884 (0.4)	185 (0.4)	1,986 (0.9)	745 (0.7)	273 (0.9)	247 (0.9)
Metastatic cancer	71 (0.3)	52 (1.0)	3,702 (1.8)	895 (2.1)	7,781 (3.4)	2,492 (2.5)	864 (3.0)	641 (2.2)
Solid Tumor without metastasis	223 (0.9)	117 (2.3)	10,394 (5.0)	2,178 (5.2)	27,014 (11.8)	7,157 (7.0)	3,884 (13.3)	2,240 (7.7)
Rheumatoid arthritis/collagen vascular diseases	155 (0.6)	128 (2.5)	1,796 (0.9)	936 (2.2)	3,318 (1.5)	2,785 (2.7)	432 (1.5)	801 (2.7)
Coagulopathy	327 (1.3)	152 (3.0)	3,367 (1.6)	732 (1.8)	7,711 (3.4)	2,782 (2.7)	1,352 (4.6)	1,209 (4.1)
Obesity	4,636 (18.6)	1,169 (23.0)	42,342 (20.3)	10,415 (24.9)	41,686 (18.2)	20,289 (20.0)	2,279 (7.8)	2,995 (10.3)
Weight loss	340 (1.4)	142 (2.9)	5,402 (2.6)	1,371 (3.3)	16,077 (7.0)	9,944 (9.8)	5,079 (17.4)	8,382 (28.7)
Fluid and electrolyte disorders	805 (3.2)	269 (5.3)	9,469 (4.6)	2,540 (6.1)	22,455 (9.8)	13,381 (13.2)	5,624 (19.3)	8,517 (29.2)
Blood loss anemia	106 (0.4)	103 (2.0)	2,110 (1.0)	698 (1.7)	6,280 (2.7)	3,544 (3.5)	1,346 (4.6)	1,861 (6.4)
Deficiency anemia	210 (0.8)	221 (4.3)	3,710 (1.8)	1,531 (3.7)	11,280 (4.9)	7,599 (7.5)	2,629 (9.0)	4,048 (13.9)
Alcohol abuse	1,909 (7.7)	242 (4.7)	15,115 (7.3)	1,707 (4.1)	8,375 (3.7)	1,393 (1.4)	397 (1.4)	164 (0.6)
Drug abuse	918 (3.7)	103 (2.0)	1,092 (0.5)	174 (0.4)	261 (0.1)	171 (0.2)	32 (0.1)	53 (0.2)
Psychoses	239 (1.0)	30 (0.6)	1,011 (0.5)	280 (0.7)	834 (0.4)	739 (0.7)	125 (0.4)	269 (0.9)
Depression	1,013 (4.1)	474 (9.3)	7,853 (3.8)	3,840 (9.2)	8,576 (3.7)	9,478 (9.3)	1,814 (6.2)	4,214 (14.4)

On average, comorbid conditions were more frequent in women (3.5) than in men (3.0). Moreover, 11.5% of men (vs 7% of women) did not have comorbidity. Patients having more than 4 comorbidities were 34% in men and 43% in women. The more the patient is older the more comorbid conditions he has (on average 1.7 for the <45 years old versus 4.1 for the >85 years old). Of note, 27% and 3.5% of the <45 years old and the >85 years old, respectively did not have comorbidity. More than half of the <45 years old had more than 4 related diagnoses compared to the <45 years old.

### Burden and trends of MI/PCI hospitalisations

#### Annual numbers

Table 1 shows that, on average, women were older than men. The variation rate between 2009 and 2014 displayed an increase in the average age in men and, conversely, a decrease in women.

The hospitalisation trends from 2009 to 2014 grew steadily with a sharp increase between 2009 and 2010. Table 3 indicates annual numbers and rates by sex. From 2009 to 2014, between 120,000 and 169,000 annual hospitalisations for MI/PCI were recorded, representing between 101,000 and 140,000 patients per year. Around two-thirds of hospitalisations were in public hospitals. The distribution of stays by type of principal diagnosis showed an increase in AMI between 2009 and 2014 for both men and women. In contrast, the number of stays decreased for OIHD.

**Table 3:** Annual numbers and standardised rates of hospitalised patients from 2009 to 2014 in metropolitan France. AMI: Acute Myocardial Infarction (ICD-10 code: I21)—OIHD: Other Ischaemic Heart Diseases (ICD-10 codes: I22 to I24)—PCI only i.e without AMI or OIHD.

	2009	2010	2011	2012	2013	2014	Global rate(%)
**Number of patients**							
**AMI**							
Men	28,628	35,096	35,432	37,624	38,509	39 643	38.6
Women	13,493	16,587	16,613	17,113	17,030	17,791	31.9
**OIHD**							
Men	5,645	5,955	6,644	6,027	5,960	5,410	-4.1
Women	2,273	2,325	2,473	2,266	2,114	1,994	-12.2
**PCI only**							
Men	44,102	54,209	54,361	57,493	59,097	64,286	45.9
Women	13,548	16,378	16,666	17,573	17,866	19,591	44.7
**Global**	101,497	122,688	124,002	129,552	131,623	139,286	37.3
**Mean age**							
**AMI**							
Men	64.1	63.9	63.9	63.9	64.0	64.1	-0.1
Women	74.5	74.6	74.2	74.2	73.9	73.9	-0.9
**OIHD**							
Men	66.0	66.0	66.0	66.4	66.4	66.8	1.4
Women	72.9	73.4	72.5	72.7	72.9	73.1	0.5
**PCI only**							
Men	65.7	65.8	66.0	66.1	66.3	66.6	1.3
Women	71.3	71.7	71.5	71.7	71.7	71.9	0.7
**Global**	67.3	67.4	67.3	67.4	67.5	67.8	0.6
**Number of stays**							
**AMI**							
Men	33,585	41,072	41,095	43,352	44,209	45,585	35.8
Women	15,601	19,175	19,009	19,556	19,441	20,337	30.6
**OIHD**							
Men	6,129	6,358	7,177	6,494	6,580	5,886	-3.9
Women	2,402	2,430	2,609	2,372	2,258	2,140	-10.8
**PCI only**							
Men	48,497	60,352	60,752	64,664	66,632	72,476	49.4
Women	10,144	11,767	13,234	12,924	13,181	14,653	47.1
**Global**	120,960	147,407	148,929	155,810	158,863	168,152	39
Hospitalisation standardised rates* (**per** 10^4^)							
**AMI**							
Men	15.0	18.2	18.1	19.0	19.3	19.8	32.1
Women	6.3	7.7	7.6	7.8	7.7	8.0	26.9
**OIHD**							
Men	2.7	2.8	3.2	2.9	2.9	2.6	-3.7
Women	1.0	1.0	1.0	0.9	0.9	0.8	-20
**PCI only**							
Men	21.7	26.8	26.8	28.4	29.1	31.5	45.2
Women	6.0	7.3	7.3	7.7	7.8	8.6	43.5
**Global**	25.7	31.1	31.3	32.5	33	34.7	35.4

* French metropolitan population.

**Fig 1:**
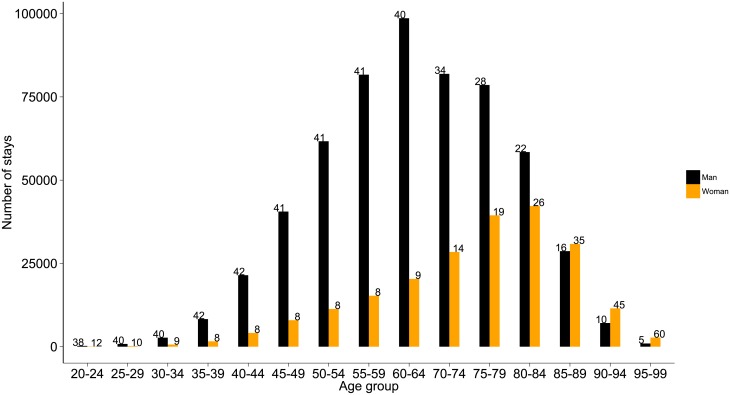
Distribution of hospital stays by sex and age group. Numbers indicate the standardised percentages.

When all ages were combined, men’s stays accounted for about three-quarters of all hospitalisations; the sex distribution differed across age groups (see Fig 1). Among the 25-64 year group, hospitalisations occurred 4.5 times more frequently in men than in women. For the 80-84 year old patients, the differences according to sex were lower: 26% in women and 22% in men. From the age of 85, hospital stays for MI/PCI were more common in women than in men.

#### Standardised rates by sex and age

As reported in Table 3, overall hospitalisation rates were significantly different (p < 2.2e-16) according to sex (39.4 per 10^4^ men and 13.3 per 10^4^ women). The AMI trend grew over the study period: 15 per 10^4^ in 2009 to 19.8 per 10^4^ in 2014 for men and 6.3 per 10^4^ to 8 per 10^4^ for women. The average rate over the entire study period was 23.7 per 10^4^ inhabitants per year.

**Fig 2:**
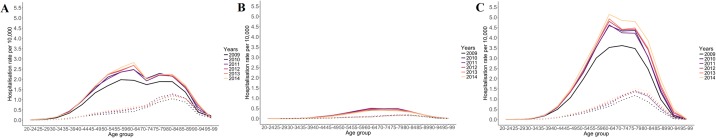
Hospitalisation rates per 10^4^ by age group and sex (dotted line for women and solid line for men) according to year. (A) Hospitalisation for AMI (ICD-10 code: I21). (B) Hospitalisation for OIHD (ICD-10 codes: I22 to I24). (C) Hospitalisation for PCI only i.e without AMI or OIHD.

When age was taken into account, there were still differences according to sex (Fig 2). As seen in Table 3, the rates were higher for PCI only, then AMI and OIHD. In women, the rates were systematically lower than the rates in men from the age of 30. For men, hospitalisation rates increased with age, peaking at 5, 2.5 and 0.5 per 10^4^ for PCI only, AMI and OIHD, respectively. in the 60-64 and 75-79 age groups, respectively. Between the ages of 60 and 70, rates decreased, except for PCI only in 2009 where the rate remained constant. For women, hospitalisation rates increased with age, peaking at 1 for AMI/PCI and at 0.15 for OIHD, respectively in the 75-84 year age group. As expected, beyond 90 years old, hospitalisation rates declined.

#### Standardised rates by region and by sex

**Fig 3:**
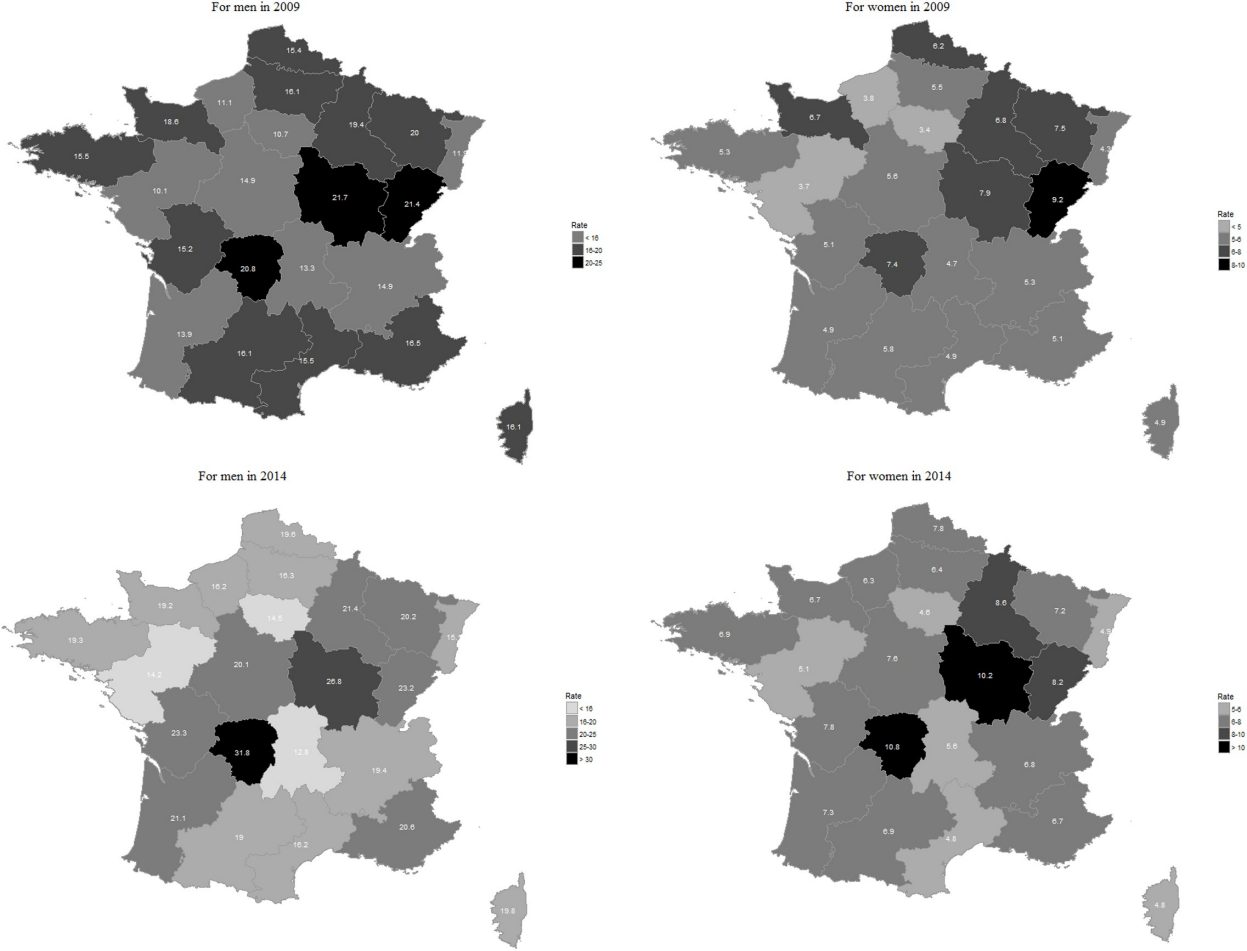
AMI hospitalisation standardised rates per 10^4^ inhabitants, by region and sex, in 2009 and 2014.

We compared results for hospitalisation standardised rates per 10^4^ inhabitants between 2009 and 2014 according to sex and region for AMI (Fig 3 and S2 Table).

For men, the average national standardised rates were 15.5 and 19.6 per 10^4^ respectively in 2009 and 2014. Per region, rates varied from 10.1 per 10^4^ in the Pays de Loire in 2009, to 31.8 per 10^4^ in Limousin, in 2014. The greatest increase of rates was noted in Poitou-Charentes (34.8%) and in Limousin (34.6%), but these regions had low population variation rates. The lowest change rate (-3.9) was detected in Auvergne.

For women, the average national standardised rates were 5.6 and 6.9 per 10^4^ in 2009 and 2014, respectively. In 2009, rates varied from 3.4 per 10^4^ in Ile de France to 10.8 per 10^4^ in the Limousin region in 2014. Changes in percentages of standardised rates between 2009 and 2014 were greatest for the regions of Haute-Normandie (39.8%) and Poitou-Charentes (34.2%), while the growth rate of the population between 2009 and 2014 was 0.3% and 0.4%, respectively. However, the lowest population change rate -13% was observed in Franche-Comté, the population of which increased by 1.3% over the six-year study period.

Standardised rates for non-AMI hospitalisations are presented in S2 Fig. The highest rates are observed in the southern and northeastern regions, regardless of the year and gender.

#### Length of hospital stay

**Fig 4:**
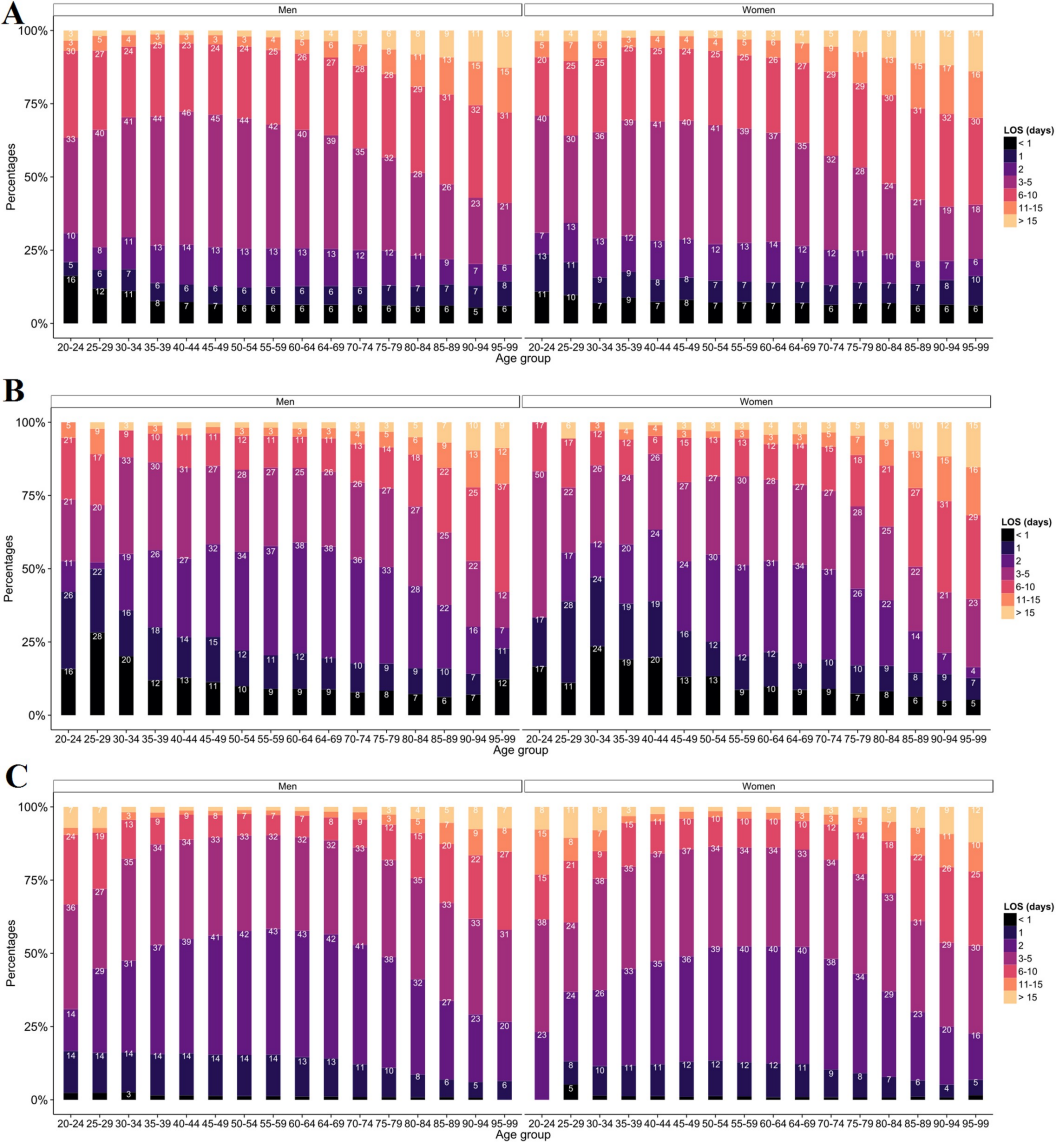
Distribution of length of stay (LOS) according to sex and age groups. (A) Hospitalisation for AMI (ICD-10 code: I21). (B) Hospitalisation for OIHD (ICD-10 codes: I22 to I24). (C) Hospitalisation for PCI only i.e without AMI or OIHD.

Most stays for AMI lasted less than 6 days (Fig 4A). In AMI, the lengths of stay (LOS) were quite similar between men and women. However, longer stays were generally observed among men. For OIHD (Fig 4B), the proportion of 2-day stays was higher for women than for men except for 25-29 years old. In PCI only stays, (Fig 4C), we observed a high proportion of long stays (> 11 days) among women less than 35 years old. In addition, among women aged 35 to 80, the proportion of 2-day stays remained relatively constant (around 10%).

In all cases combined, the LOS varied substantially: 3.7% of stays lasted less than 1 day, 9.4% lasted 1 day, whereas the 2 and 3-5 days concerned 27.4% and 33.5% of hospital stays, respectively. Only 3.5% of hospitalisations lasted more than 15 days. The LOS increased with age. The average LOS in the 20-24, 55-59 and +90 year of age groups was 5, 3 and 7 days, respectively. Stays of 11-15 days represented 4% of stays for the 20-24 years group, and 16% of stays for the 95-99. In 2014, the average of AMI LOS was 5.7 days, and was significatively lower (p < 9.8e-13) than in 2009 (6.0).

#### Hospital costs

The evolution of hospital costs over the observation period is presented in Table 4. In line with the evolution of the number of stays and patients (Table 3), there is a constant increase in direct costs from 2009 to 2014, except for OIHD, with a rate of change of about 42%. In contrast, for OIHD, we observed a decrease of about 3%. In France, the economic burden of CAD was € 3.65 billion in 2009 compared with € 5.08 billion in 2014.

**Table 4:** Estimated hospital costs* (€ billion) of AMI, OIHD and PCI only admissions in France from 2009 to 2014 with the overall evolution rate.

	2009	2010	2011	2012	2013	2014	Global rate (%)
AMI	1.43	1.79	1.81	1.96	1.95	2.03	41.8
OIHD	0.22	0.22	0.25	0.22	0.23	0.21	-3.3
PCI only	2.01	2.42	2.45	2.62	2.62	2.85	41.7

* hospital costs were estimated using hospital tariffs.

### Burden and trends of MI/PCI readmissions

#### Readmission rates

**Fig 5:**
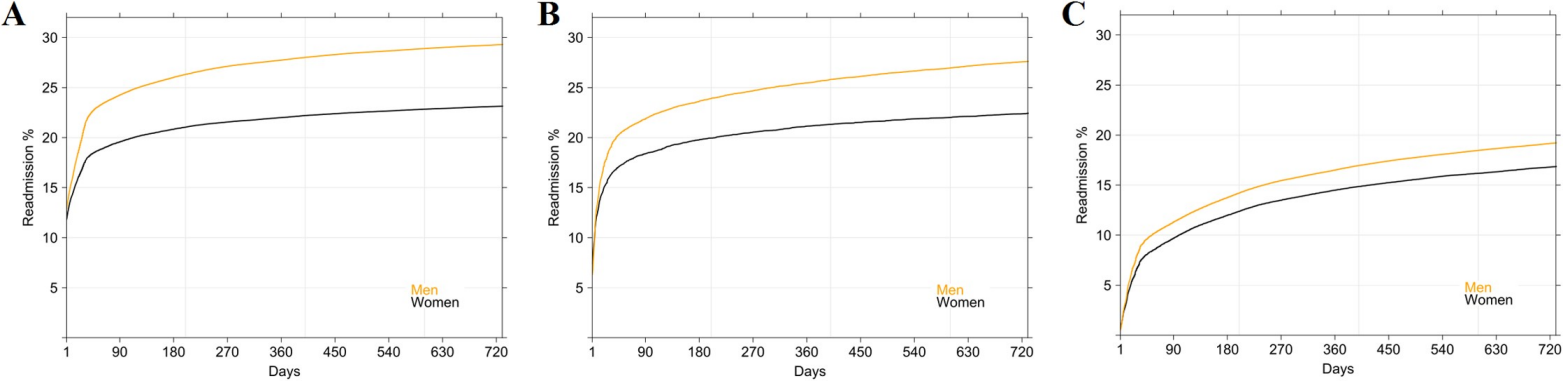
Readmission rates: Kaplan-Meier analysis by sex. (A) When first admission was AMI (ICD-10 code: I21). (B) When first admission was OIHD (ICD-10 codes: I22 to I24). (C) When first admission was PCI only i.e without AMI or OIHD.

The median number of readmissions was one; 78% were readmitted twice, 18% thrice and 4% four fold or more. Men were significantly more readmitted than women (p < 2.2e-16) whatever the reason of first admission (Fig 5). Readmission rates were different according to the reason of first admission: rates were higher for AMI, then OIHD and PCI only. For instance, when first admission was AMI, the readmission rates by sex varied between 17 and 21% at month 1, between 20 and 24% at month 3, between 21 and 26% at month 6, between 22 and 28% at year 1 and between 23 and 29% at year 2.

**Fig 6:**
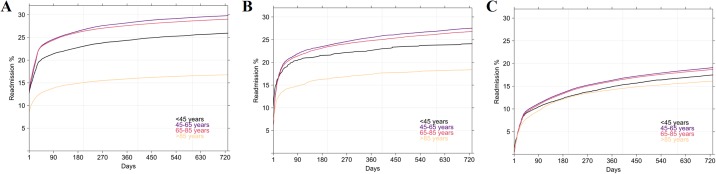
Readmission rates: Kaplan-Meier analysis by age group. (A) When first admission was AMI (ICD-10 code: I21). (B) When first admission was OIHD (ICD-10 codes: I22 to I24). (C) When first admission was PCI only i.e without AMI or OIHD.

Fig 6 shows the readmission rates by age. Patients over 85 years had significantly lower readmission rates (p < 2.2e-16) whatever the reason of first admission. However, this observation is more important when AMI was the first admission than PCI only for example. Besides, The gap between the readmission rates of the 45-65 age group and the over 85 age group was larger for AMIs than for non-AMIs. AMI readmission rates by age ranged between 12 and 21% at month 1, between 14 and 25% at month 3, between 15 and 26% at month 6, between 15 and 27% at month 9, between 16 and 28% at year 1 and between 17 and 30% at year 2. As in Fig 5, readmission rates were higher for AMI, then OIHD and PCI only.

#### Readmission time

Analysing only readmissions, the average number of hospital stays during the 2009-2014 period was 2.3 (95% confidence interval [CI]: 2.29-2.32) with a significant difference (p < 2.2e-16) between men and women. Among patients who where rehospitalised, some (41,224 women and 129,677 men) were hospitalised several times in the six-year period, representing a total of 353,970 stays (85,867 for women and 268,112 for men). The median time until rehospitalisation with I21 ICD-10 code as primary diagnosis, was 41 days (average 208 days (95% CI: 206-212). Among rehospitalised patients, it occurred within three months in half of all cases. The readmission time was significantly different (p < 6.25e-06) between men and women, occurring on average 11 days later in men. The median readmission time was three months for both groups.

Table 5 summarises readmission times according to sex in AMI and non-AMI. For AMI, a relapse occurred within 48 hours, more often for women (6.2%) than for men (4.8%). A readmission was observed beyond six months in 38.2% of cases (38.4% for men vs. 37.6% for women). For non-AMI, the percentages of readmissions within 3 days are low (less than 2%). Beyond 6 months, there is readmission in about 2 out of 5 cases.

**Table 5:** Distribution of readmission times according to sex during the first year.

	AMI						non-AMI					
	Men		Women		Total		Men		Women		Total	
	n	%	n	%	n	%	n	%	n	%	n	%
<24h	1,078	2.91	445	4.19	1,523	3.19	209	0.26	80	0.42	289	0.29
24h	294	0.79	93	0.88	387	0.81	309	0.39	77	0.40	386	0.39
24-48h	39	1.05	117	1.10	508	1.07	689	0.87	169	0.88	858	0.87
2-3 days	412	1.11	136	1.28	548	1.15	928	1.18	213	1.11	1141	1.16
3-7 days	2,031	5.48	592	5.57	2,623	5.50	5,077	6.43	1,048	5.45	6,125	6.24
7-15 days	4,389	11.84	1,172	11.03	5,561	11.66	7,785	9.87	1,808	9.40	9,593	9.78
15-30 days	7,573	20.43	1,951	18.37	9,524	19.97	9,572	12.13	2,228	11.59	11,800	12.02
1-3 months	6 448	17.39	1,892	17.81	8,340	17.49	10,871	13.78	2,865	14.90	13,736	14.00
3-6 months	3,745	10.10	1,162	10.94	4,907	10.29	9,670	12.25	2,577	13.41	12,247	12.48
6-12 months	3,886	10.48	1,122	10.56	5,008	10.50	11,434	14.49	2,934	15.26	14,368	14.64
>1 year	6,826	18.41	1,939	18.26	8,765	18.38	22,367	28.34	5,225	27.18	27,592	28.12
**Total**	37,073	99.99	10,621	99.99	4,694	100	78,911	99.99	19,224	100	98,135	99.99

#### Regional disparities

We compared readmission rates at 3 months, 9 months and one year for AMI and non-AMI (S3 Fig). There is a little asymmetry; readmission rates according to region are different according to the sex. Men rates were higher than women, particularly in AMI readmissions. In addition we observed a North-South readmission divide, except Burgundy.

#### Description of care pathways according to hospital diagnosis

**Fig 7:**
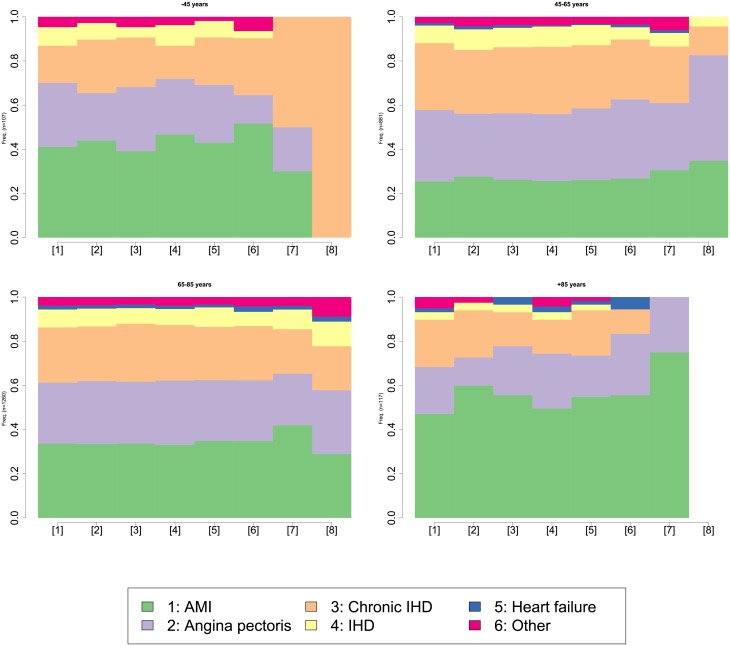
Cross-sectional distributions of principal diagnosis by age for patients who experienced 4 to 8 hospitalisations during 2009-2014.

Sequences of events were analysed by sex and age. Data were separated according to length of sequences (i.e. number of hospitalisations in the study period for a given patient). The first analysis carried out on short length sequences (less than 5 events) did not highlighted between-group differences. A further analysis carried out on long sequences (at least 5 events, and less than 8 events) gave different results. Trajectories were significantly different by sex (p < 0.01) but also by age (p < 0.02). The chronogram, displayed in Fig 7, is a status distribution plot. It represents the general patterns of the whole set of sequences by age group. Fig 7 shows jumps in the sequence of the principal diagnosis distributions, more specifically between the <45 and the 45-65 year group and between the >85 and 65-85 year group from the sixth event onward. AMI was more frequent in women but also in the <45 and the >85 years, irrespective of sex.

### Burden of in-hospital mortality

#### In-hospital mortality by sex and age

**Fig 8:**
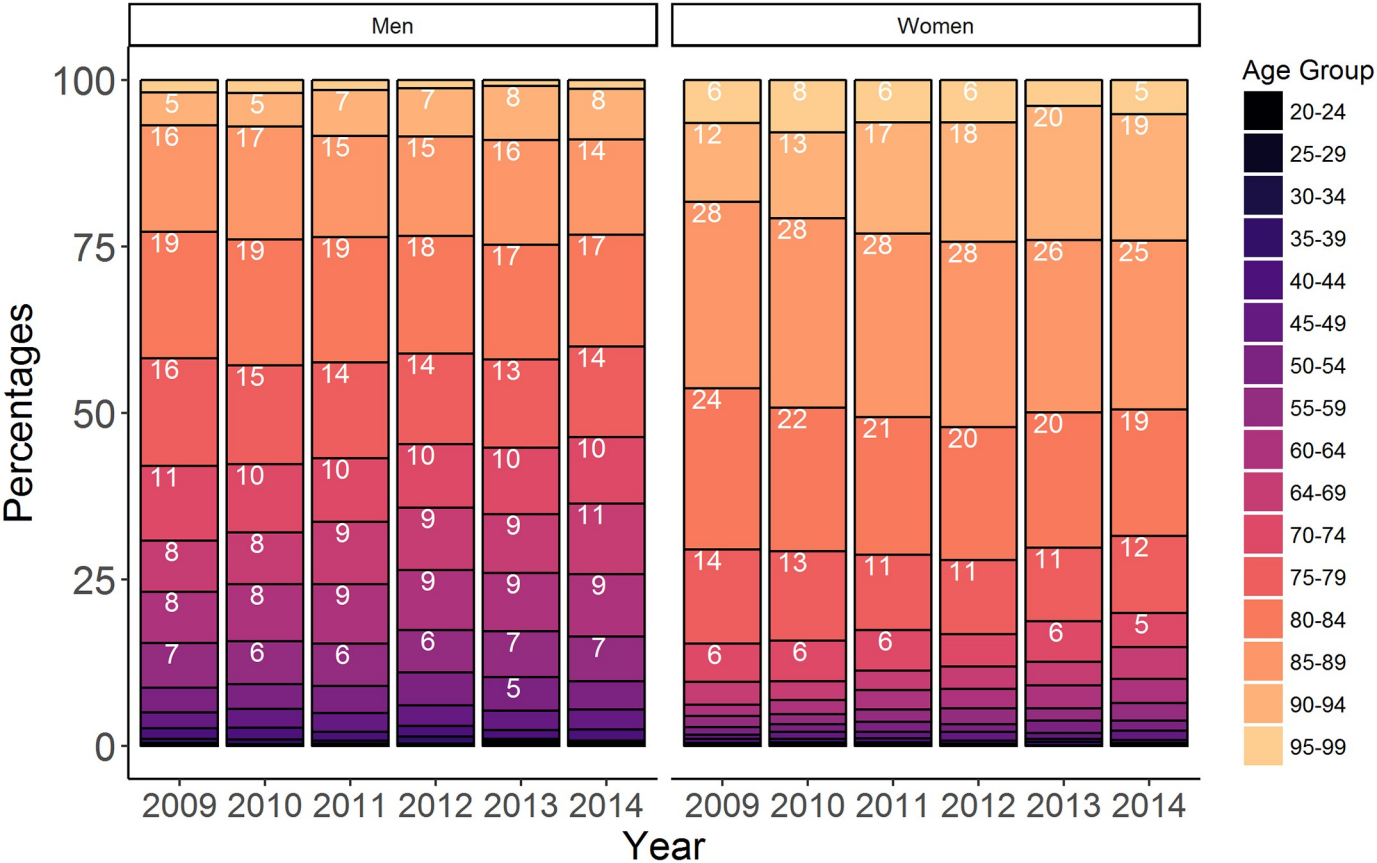
Proportion of in-hospital deaths by age group according to sex and year. For sake of readability, proportions were only displayed for values greater than or equal to 5%.

Fig 8 represents the proportion of deaths according to age group by sex and year. Over the six-year period, regardless of sex, in-hospital fatalities mostly affected the 80-89 age group with a rate of 20%. In women, the proportion of deaths was higher in the 80-94 age group, with percentages ranging from 19% to 28% depending on the years. Whereas in men, the proportion increased with age and decreased over 90-year old.

#### In-hospital mortality rates

In the years 2009 to 2014, 3.3% (29,728 subjets) of hospitalised patients died with an in-hospital fatality rate ranging from 80 to 95 per 10^4^ hospital stays per year. Thus, every year, between 4,212 and 5,252 people hospitalised with MI/PCI died during their hospital stay. Hospital death decreased significantly by 15% (p < 2.2e-16) during the period from 4,2% in 2009 to 3.6% in 2014. The in-hospital mortality rate was higher among women (between 0.013 and 0.022 per 10^4^ hospitalisations) than among men (0.003 per 10^4^ hospitalisations).

The analysis by age showed higher rates in patients aged less than 35, regardless of sex. The rates decreased until 40 years old, and from the 75-79 age group onwards rates grew exponentially with age.

## Discussion

### Primary and associated diagnoses

During the five years from 2010 to 2014, the number of incident cases increased regardless of the reason for hospitalisation. However, our study highlights differences according to the diagnosis at first admission during the study period. Indeed, only the incident cases of OIHD observed a decline of about 14%. Unlike PCIs, the increase in AMI incidence was greater among men. There are potential reasons for these findings: a wider access to healthcare from the first MI symptoms through information and awareness campaigns, together with a better dissemination of recommendations published by the French National High Authority for Health since 2007 following the consensus conference of November, 2006 [27]; an increase in MI cases, although the health survey in 2015 found no change in MI cases in the French population from 2000 to 2010 [28]; a slight increase in risk factors was observed, for instance, daily smoking prevalence in France between 2005 and 2010 increased 7% [28]; and finally the definition used in this analysis is less precise than those of the Third Universal Definition of Myocardial Infarction [29].

Globally, our analysis confirmed a preponderance of incident stays for men, already established in the literature [30], almost three times more frequent than in women. The current burden of MI/PCI is influenced by cardiovascular health disparities. The analysis of the health status of the population studied through the comorbidities of the Elixhauser score shows that a significant proportion of the population suffers from cardiovascular disorders, hypertension, obesity and diabetes. This observation, explained by the evolving change in lifestyle (tobacco use, decreased physical activity, and westernization of traditional diets), is worrisome and contributes to the burden of hypertension, an important contributor to CAD [31]. This is how the fight against the progression of the CAD epidemic is deeply linked to that of the progression of obesity and sedentary lifestyle [32]. In addition, modifiable risk factors must be added to that of air pollution. In view of this work, it would be interesting to complete this work by considering the environment aspect with data on air quality measurements [33].

### Burden and trends of MI/PCI hospitalisations

During the 2009-2014 period, hospitalisations for MI/PCI represented an annual average of 150,000 stays and an average of about 125,000 patients in health services. We highlighted that the annual hospitalisation trend was globally increasing. Meanwhile, the total number of hospital stays for any reason decreased from 11.96 million in 2009 to 11.31 million in 2014. Therefore, MI/PCI hospital burden has increased over the study period, representing 1% of all stays in 2009 compared to 1.5% in 2014.

Our study reported increasing rates in young women. However, the latter may be driven by the low overall frequency, making a small absolute increase account for a high relative change. We also observed a difference according to age groups: women were affected later in life, together with a higher life expectancy (84.4 years for women vs. 77.7 years for men in 2009, source Insee 2015), and an aging population between 2009 and 2014 (About 57%, source Insee 2016).

In the present data, AMI cases were heterogeneous across the country. High hospitalisation rates in the South were due to elderly population growth in these regions. At a national level, the MONICA project highlighted an MI case geographic disparity worldwide, but also within each country, with a North-South divide [34].

In most cases, the LOS depended on patients’ age and MI complications. We found that for elderly patients the average LOS was seven days, versus three days for patients between 55 and 59 years old. This is consistent with the French average LOS of one week. Early hospital discharge is possible: in an uncomplicated revascularized MI, the vital risk drops significantly at three days [34]. The MI care is different according to countries: it is four weeks in Japan [35], four days in Norway and Denmark, about 10 days in Germany and Greece according to the OECD (Organisation for Economic Co-operation and Development) survey [36].

From an economic point of view, the gross cost of hospitalisation is increasing during the observation period for AMIs and PCIs and decreasing for OIHD in line with hospitalisations trends. The cost of a hospital stay may vary from one patient to another, depending on LOS, age and comorbid conditions. In average, CAD hospital cost was around 4 billion euros [5]. However, since it is difficult to determine the accountability of comorbidities, to refine these results, it would be interesting to take into account both all hospitalisations of patients (not only those related to CAD) but also the outpatient care consumption [37].

### Burden and trends of MI/PCI readmissions

In summary, our analysis highlighted several key points which might be helpful in patient care optimisation and healthcare resources allocation. Firstly, readmission for an MI/PCI occurred in 30% of patients and was more frequent in men and in the 65-85 age group. This confirmed the discrepancies in risk linked to sex and age group [38]. Secondly, readmission rates were highest for, in that order, an AMI, an OHID and a PCI, whatever the diagnosis of the first admission. Thirdly, in patients with AMI, 14% were readmitted within the first month and 16% within the following two-to-three months. These results are quite similar to those of one study which established that 20% of patients with a primary MI diagnosis were readmitted or died one month later. Moreover, the analysis of the readmission time revealed that 75% of MI relapse occurred in the year following the primary diagnosis [39]. Fourthly, we highlighted a South-North divide in readmissions. Regional variability in population MI incidence and mortality was ascertained in Spain [40], and in other countries by the WHO-MONICA Project [9]. Besides, a sex discordance between certain regions where hospitalisation rates for men were always higher, as was incidence in females was established. Yet mortality rates were not abnormally elevated, except in Corsica.

As we took into account all readmissions without distinguishing scheduled readmissions (for instance, an angioplasty for another vessel) from those resulting from an evolution of the disease, our analysis has established a consistent readmission burden for MI/PCI for both recurrences and follow-up care after a first episode. Moreover, with regard to care follow-up, the sequence analysis revealed that regardless of the timing of the care pathway, AMI diagnosis was most common in women, but also in the extreme age groups (<45 years and >85 years). Conversely, in the middle age groups, there were more events such as CIHD and IHD. These results are consistent with the US study of readmission rates after AMI by age and sex [38]. In addition, we noticed that some patients had similar pathways. This information could prove invaluable for health planning: adapting the patient’s care pathway or better managing the availability of resources. Based on this observation, we investigate an extension work. We have developed a model to characterize hospital healthcare flows from patient care pathways in order to improve their knowledge in a perspective of possible change in public health policies for the improvement of patient care [41].

### Burden of in-hospital mortality

Our findings established a decline in annual mortality rates over the study period. These results are consistent with previous investigations and tend to suggest an improved prognosis for these patients and better management of acute coronary events, as well as risk factors in secondary prevention [5]. The latter might explain the inflation of the economic burden.

Furthermore, our results pinpointed that adjusted mortality rates were higher for inpatients aged less than 40 and more than 80, but a conclusive link with comorbidity could not be found. In fact, administrative data are not designed to explore this question. Death generally occurred during the first hospital admission [42]. Moreover, this risk of death was higher in women. However, women generally suffered MI later than men. Thus, they were older at MI occurrence which may contribute to a higher severity of a subsequent cardiac event.

Most countries referred to nationwide clinical registries to evaluate and forecast trends in MI [7, 43, 44]. AMI mortality rate was assessed at 9.7% in Great Britain with National Institute for Cardiovascular Outcomes Research/Myocardial Ischaemia National Audit Project and CArdiovascular disease research using LInked Bespoke studies and Electronic health Records, at 8.4% in Sweden with Swedish Web-system for Enhancement and Development of Evidence-based care in Heart disease Evaluated Accorded to Recommended Therapies/Register of Information and Knowledge about Swedish Heart Intensive care Admissions, at 10.8% in Germany with hospital data and at 5.3% in France with FAST-MI (French nationwide registry). Nevertheless, health coding systems, MI case identification algorithms and years studied did not allow comparison of the reported rates with our results.

In future work, we plan to elaborate a prognostic model of in-hospital mortality in acute coronary syndrome from patient pathways to predict fatality risk. Indeed, a better knowledge of the relationship between care pathways, associated with chronic conditions and mortality might help to combat this public health issue by reducing CAD mortality burden.

### Data completeness

Although we attempted to provide a systematic view in annual MI burden, our investigation underlined limits in data completeness due to the retained choice of the MI case identification algorithm.

Patients with MI not seen by the healthcare providers can be categorised in two groups: 1) Silent MI without health care [45]. The patient are unaware they have had a heart attack since they did not experience the classic symptoms such as chest pain; 2) Sudden cardiac death. Indeed, most of the deaths after MI occurred outside health facilities: of the 33,435 annual deaths (Source Center for Epidemiology on Medical Causes of Death 2013), circa 5,085 deaths occurred during hospitalisation (Source ATIH 2013).

Some patients, out of hospital after emergency services, probably considered the least serious or on the contrary who died in the emergency room, are not recorded in the PPS database. In this instance, confining the analysis to hospitalised MI in PPS gives a homogeneous but partial view of the MI burden.

Several studies compared cardiac registers with medico-economics data. For instance, a MI monitoring study [21] reported a sensitivity rate about 76% and positive predictive value (PPV) of 78% in detecting this pathology with ICD-10 in PPS, regardless of age between 35 and 74, and irrespective of gender. Another study used ICD-10 codes based on data from 2004 to 2008, comparing accuracy in detecting MI between PPS and register obtained sensitivity of 77% and PPV of 69% [20]. At the international level, there is an investigation on validation of identification MI algorithms from electronic healthcare records in different countries using distinctive disease coding system [18]. The identification algorithms based on ICD codes in Italy, Denmark and the Netherlands had a PPV greater than 90%. Moreover, Coloma et al. suggested that using several sources of information offers diverse perspectives and complementary knowledge. For instance, GPs databases when available. Hospital base report information related to care, such as stenting or intraluminal dilatation, was provided in health facilities only.

## Conclusion

We quantified the burden of CAD, as opposed to other studies estimating changing trends, this requires taking into account the entire consumption of hospital care. Our findings, based on 900,121 stays representing 678,021 patients with MI/PCI during the study period, showed that the burden of CAD is persisting making it a major public health issue exerting heavy economic costs. Indeed, we underscored a constant increase in AMI and PCI burden meaning rising hospitalisation rates with rising health care costs. In contrast, we observed a decline in OIHD burden. However, mortality rates were decreasing illustrating the medical progress in reducing CVD fatality in recent decades, but also the positive impact of health campaigns to address risk factors such as smoking bans in work and public places [46] and reducing salt intake [47].

Although our results are to be modulated because of the limits in PPS data completeness, these data appears as a complementary approach to follow up the burden of AMI hospitalisations but also for for public health surveillance of several conditions. Moreover, these data can be completed with information collected by the French health insurance system [48] from medico-economic research.

Given the demographic changes (growing population, ageing population…) with the still rising risk factors, the future CAD burden is expecting a further increase. Therefore, it is essential to continue monitoring and measuring the burden of this epidemic in order to measure the impact of anti-development campaigns, as well as to anticipate the real needs for the years in terms of staff and infrastructure.

## Supporting information

S1 Table Codes of the ICD-10th and the French CCMP for the MI/PCI case identification algorithm in the PPS databases. (PDF)

S2 Table Distribution of French inhabitants by regions according to sex and average annual growth rate from 2009 to 2014. (PDF)

S1 Fig Flow chart of data selection. (TIFF)

S2 Fig Non-AMI hospitalisation standardised rates per 10^4^ inhabitants, by region and sex, in 2009 and 2014. (TIFF)

S3 Fig Readmission time after 3, 9 and 12 months by sex and region (from South to North). (A) When admission was AMI (ICD-10 code: I21). (B) When admission was non-AMI.
(TIFF)
