# Supplementary material for: Hospital burden of coronary artery disease: Trends of myocardial infarction and/or percutaneous coronary interventions in France 2009–2014
Source: PLoS One. 2019 May 2;14(5):e0215649. doi: 10.1371/journal.pone.0215649 (PMC6497251; doi:10.1371/journal.pone.0215649)
Supplement: S1 Table — (PDF) [file pone.0215649.s001.pdf]

*S1 Table. Codes of the ICD-10th and the French CCMP for the MI/PCI case identification algorithm in the PPS databases.*

**International Classification Diseases 10th version Codes**

| Code | Detail |
|---|---|
| I21 | Acute myocardial infarction |
| I22 | Subsequent myocardial infarction |
| I23 | Certain current complications following acute myocardial infarction |
| I24 | Other acute ischaemic heart diseases |

**Common Classification of Medical Procedures Codes**

| Code | Detail |
|---|---|
| DDAF001 | Intraluminal dilatation of a coronary vessel without stenting, transcutaneous arterial |
| DDAF003 | Intraluminal dilatation of 3 or more coronary vessels with stenting, transcutaneous arterial |
| DDAF004 | Intraluminal dilatation of 2 coronary vessels with stenting, transcutaneous arterial |
| DDAF006 | Intraluminal dilatation of a coronary vessel with stenting, transcutaneous arterial |
| DDAF007 | Intraluminal dilatation of 2 coronary vessels with coronary arteriography with stenting, transcutaneous arterial |
| DDAF008 | Intraluminal dilatation of a coronary vessel with coronary arteriography with stenting, transcutaneous arterial |
| DDAF009 | Intraluminal dilatation of 3 or more coronary vessels with coronary arteriography with stenting, transcutaneous arterial |
| DDAF010 | Intraluminal dilatation of a coronary vessel with coronary arteriography without stenting, transcutaneous arterial |
| DDFF001 | Intraluminal coronary artery atherectomy by rotational method, transcutaneous arterial |
| DDFF002 | Intraluminal coronary artery atherectomy by directional method, transcutaneous arterial |
