# Supplementary material for: Hospital burden of coronary artery disease: Trends of myocardial infarction and/or percutaneous coronary interventions in France 2009–2014
Source: PLoS One. 2019 May 2;14(5):e0215649. doi: 10.1371/journal.pone.0215649 (PMC6497251; doi:10.1371/journal.pone.0215649)
Supplement: S2 Table — (PDF) [file pone.0215649.s002.pdf]

S2 Table. Distribution of French Inhabitants by regions according to sex and average annual growth rate from 2009 to 2014.

Region	Sex	Population						Annual growth rate (%)
		2009	2010	2011	2012	2013	2014	
Alsace	M	669 965	672 813	675 957	679 163	681 876	686 458	0.49
	W	723 230	725 180	728 256	731 179	733 677	741 096	0.49
Aquitaine	M	1 170 514	1 181 478	1 190 981	1 201 315	1 210 641	1 227 326	0.95
	W	1 311 282	1 321 978	1 331 132	1 341 830	1 350 766	1 367 094	0.84
Auvergne	M	500 007	501 479	504 543	506 434	507 553	508 525	0.34
	W	549 879	550 211	551 430	552 680	553 310	555 976	0.22
Basse-Normandie	M	528 858	531 810	533 289	535 375	536 826	536 895	0.3
	W	583 318	585 734	587 881	589 885	590 885	591 106	0.27
Burgundy	M	601 664	603 454	604 544	605 427	605 872	605 261	0.12
	W	664 036	664 833	666 167	666 843	666 903	665 989	0.06
Brittany	M	1 144 204	1 154 495	1 163 362	1 171 915	1 180 203	1 189 240	0.78
	W	1 257 208	1 266 929	1 273 997	1 282 533	1 290 436	1 298 329	0.65
Centre	M	921 444	924 730	927 351	930 469	933 180	934 515	0.28
	W	1 007 264	1 010 076	1 014 855	1 019 112	1 022 087	1 024 547	0.34
Champagne-Ardenne	M	481 914	482 878	483 902	484 339	484 263	486 540	0.19
	W	526 700	526 007	527 253	527 201	526 698	529 341	0.1
Corsica	M	116 879	117 905	120 109	121 948	123 731	123 397	1.09
	W	126 043	126 918	128 978	130 402	131 959	133 923	1.22
Franche-Comté	M	425 104	426 397	427 593	429 005	430 580	431 247	0.29
	W	453 626	454 655	455 847	457 311	458 461	459 538	0.26
Haute-Normandie	M	643 469	646 288	648 614	651 055	653 239	655 311	0.37
	W	715 960	718 911	721 000	723 676	725 530	727 391	0.32
Ile-de-France	M	4 114 166	4 135 376	4 161 770	4 186 094	4 208 579	4 221 669	0.52
	W	4 571 811	4 594 713	4 620 064	4 646 085	4 671 065	4 681 564	0.48
Languedoc-Roussillon	M	940 158	950 114	962 878	974 680	986 093	997 858	1.19
	W	1 059 907	1 071 814	1 084 188	1 096 197	1 107 559	1 125 115	1.2
Limousin	M	280 041	279 712	278 865	278 713	278 342	275 891	-0.29
	W	309 830	310 260	309 513	309 402	309 129	306 326	-0.23
Lorraine	M	858 196	860 220	861 709	863 625	865 010	862 945	0.11
	W	929 882	932 356	933 387	935 155	935 850	933 775	0.08
Midi-Pyrénées	M	1 058 299	1 066 386	1 074 895	1 083 069	1 091 496	1 102 577	0.82
	W	1 150 847	1 159 415	1 167 396	1 175 445	1 182 704	1 197 360	0.79
Nord Pas de Calais	M	1 386 140	1 389 885	1 394 057	1 398 289	1 401 560	1 409 621	0.34
	W	1 556 152	1 558 821	1 562 421	1 565 348	1 567 015	1 572 252	0.21
Pays de Loire	M	1 262 526	1 274 082	1 284 579	1 295 710	1 305 727	1 317 498	0.86
	W	1 373 432	1 387 214	1 398 392	1 409 957	1 420 190	1 430 320	0.82
Picardy	M	677 930	679 285	681 571	683 476	685 148	688 019	0.29
	W	731 988	734 223	737 078	739 096	740 397	743 494	0.31
Poitou-Charentes	M	648 648	652 879	656 813	660 153	662 940	664 010	0.47
	W	715 661	719 619	722 768	726 387	728 969	731 224	0.43
PACA	M	1 751 141	1 759 245	1 769 471	1 777 398	1 784 466	1 796 756	0.52
	W	1 997 353	2 003 680	2 011 028	2 016 988	2 020 418	2 034 734	0.37
Rhône-Alpes	M	2 202 069	2 224 430	2 245 109	2 266 394	2 286 893	2 309 097	0.95
	W	2 398 124	2 419 755	2 444 943	2 466 852	2 487 557	2 511 882	0.93
Metropolitan France	M	22 383 336	22 515 341	22 651 962	22 784 046	22 904 218	23 030 656	0.57
	W	24 713 533	24 843 302	24 977 974	25 109 564	25 221 565	25 362 376	0.52

Insee source.
