# Supplementary figures and images for: Hospital burden of coronary artery disease: Trends of myocardial infarction and/or percutaneous coronary interventions in France 2009–2014
Source: PLoS One. 2019 May 2;14(5):e0215649. doi: 10.1371/journal.pone.0215649 (PMC6497251; doi:10.1371/journal.pone.0215649)

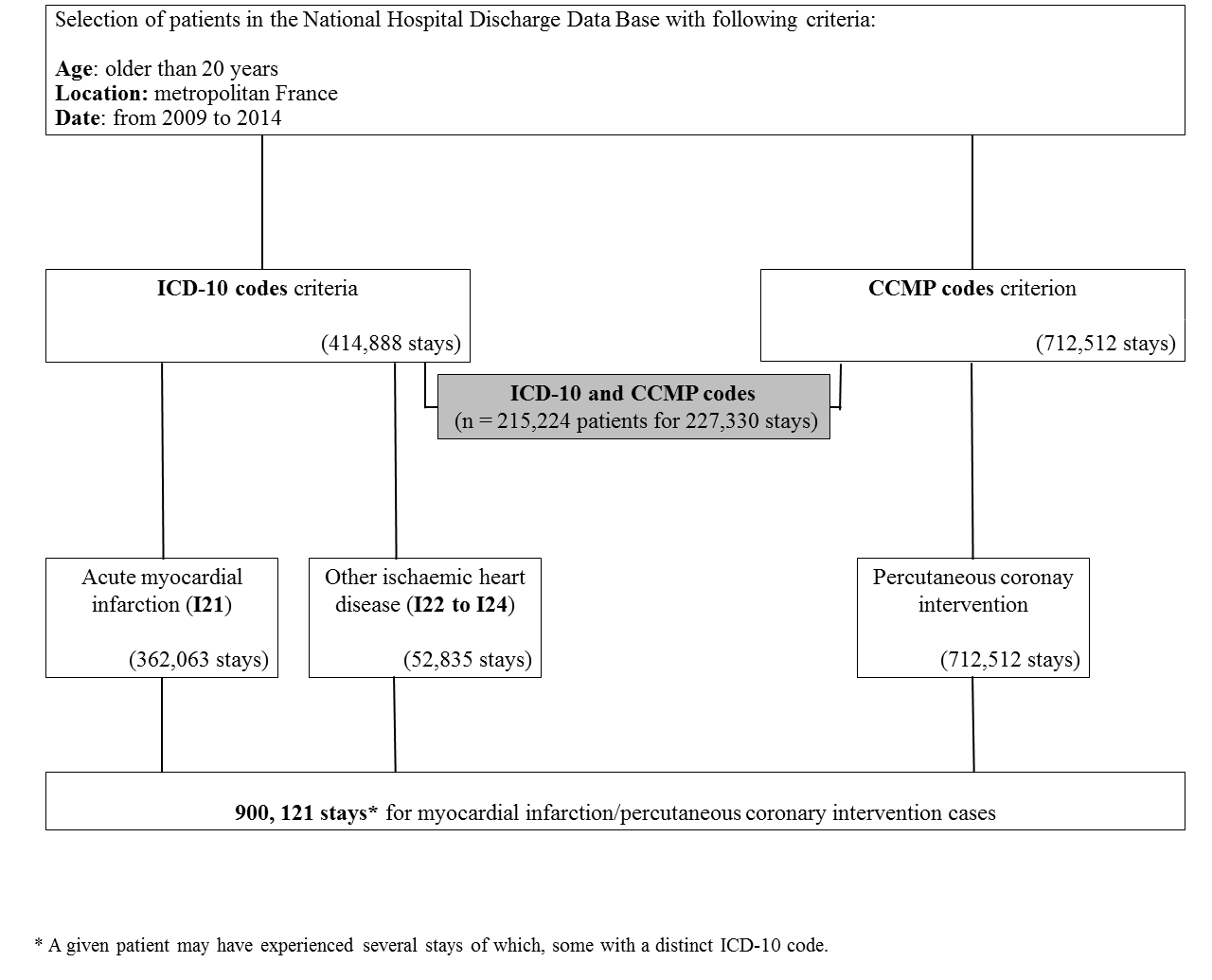

Supplement: S1 Fig — (TIFF) [file pone.0215649.s003.tiff]

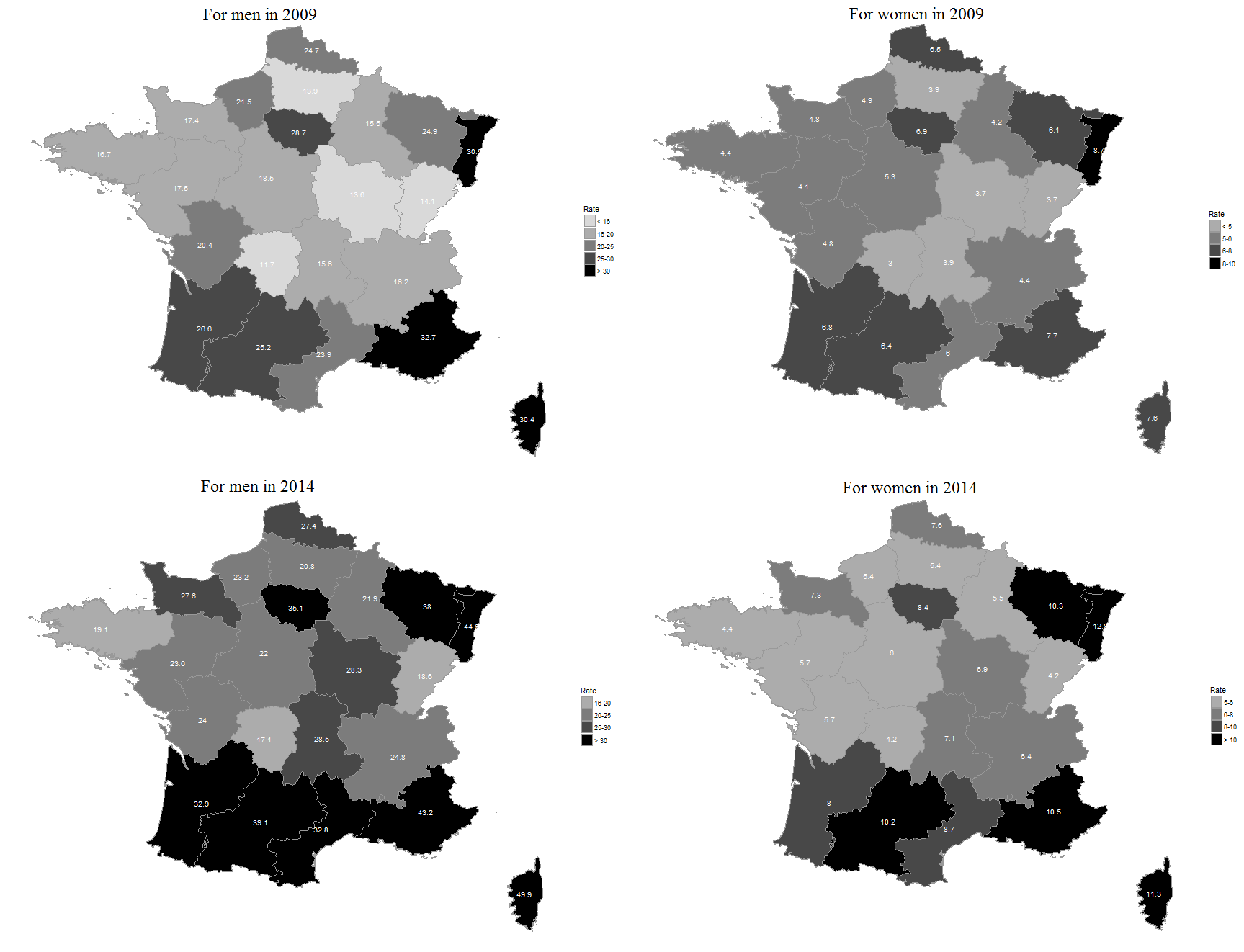

Supplement: S2 Fig — (TIFF) [file pone.0215649.s004.tiff]

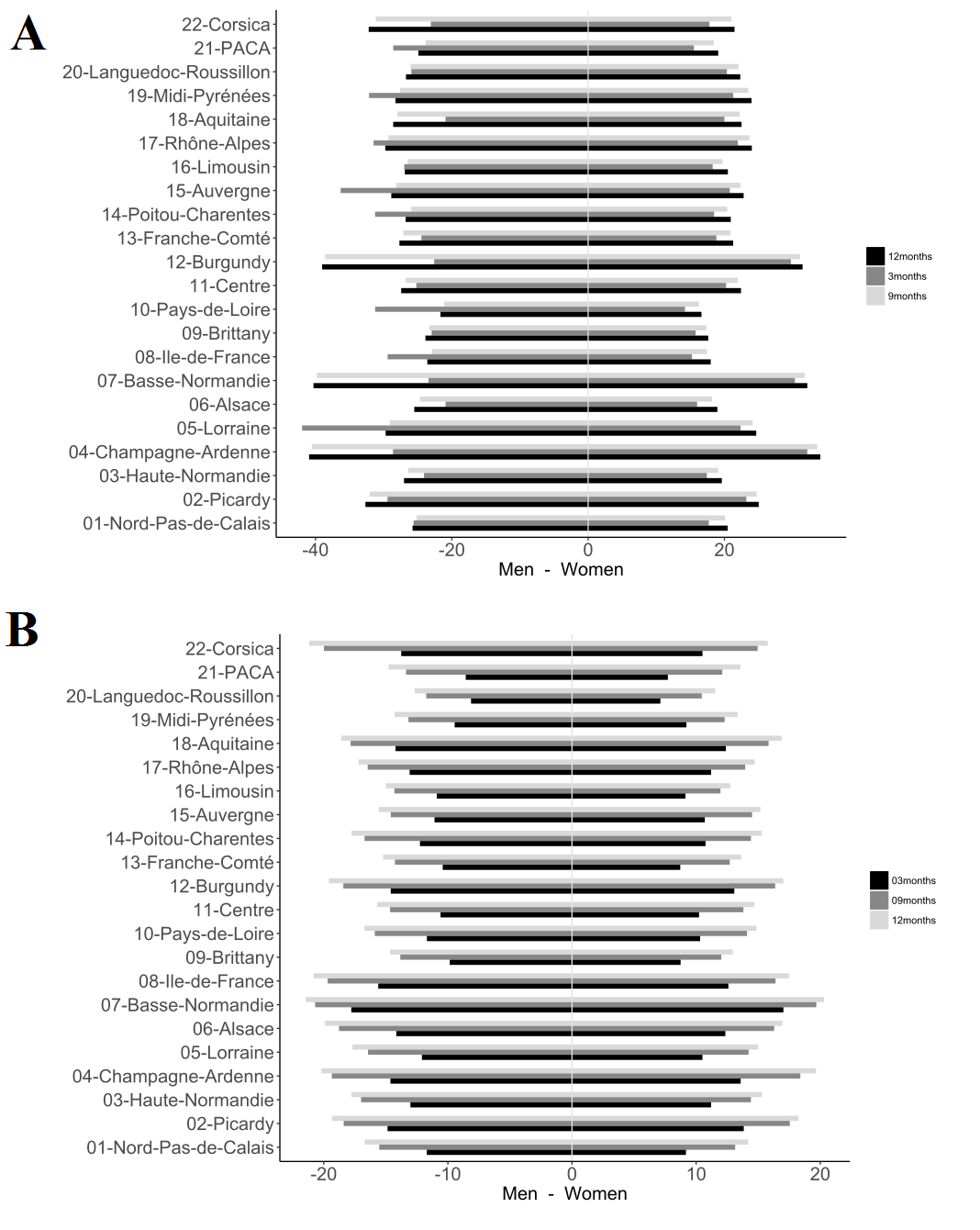

Supplement: S3 Fig — (A) When admission was AMI (ICD-10 code: I21). (B) When admission was non-AMI. (TIFF) [file pone.0215649.s005.tiff]
